# Retraction: Histones-Mediated Lymphocyte Apoptosis during Sepsis Is Dependent on p38 Phosphorylation and Mitochondrial Permeability Transition

**DOI:** 10.1371/journal.pone.0244473

**Published:** 2020-12-17

**Authors:** The PLOS ONE Editors

Following the publication of this article [1], concerns were raised regarding the flow cytometry data presented in Fig 2 and Fig 3, the sample size employed in the study, and the statistical analyses reported:

- The 0 µg/ml panel of Fig 2A and the 0 h panel of Fig 2B look similar.
- The 100 µg/ml panel and the 200 µg/ml of Fig 2A, and the 2 h panel of Fig 2B look similar.
- The GAPDH panel of Fig 3A looks similar to the GAPDH panel of Fig 4B. Raw image data were provided in support of these panels, including four independent replicates of the p-p38 experiment and two replicates of the Bcl2 experiment. These data suggest that the wrong image was reported for GAPDH in Fig 4B, and that the GAPDH panel shown in Fig 3A is not the matched internal control for the p38 and p-p38 data included in the figure.
- The sample group represented in Fig 2 and Fig 3 include only three replicates per condition, with heterogeneous variances between the four groups, raising concerns about the power of the study. A sample size computation to determine if the study is powered to test the hypothesis was not reported in the article, and a statistical advisor commented that the study was not sufficiently powered to support the conclusions drawn in the study.
- Concerns were raised about the use of parametric analyses (multiple Student t-tests) in Figs. 2 and 3 as the data did not conform to a normal distribution. The statistical advisor indicated that non-parametric tests ought to have been used instead, and that an analysis by ANOVA of the available results from the original experiments did not yield statistically significant results.

In response to the first two points, above, the authors provided comments, dot plots and quantification data, and offered replacement panels. However, their comments, data, and replacement panels did not fully resolve the concerns. The authors indicated that the raw.fcs data are no longer available for the flow cytometry experiments reported in Fig 2.

In post-publication discussions, the authors noted that they used the following formula to compute sample size: n = Ψ^2^ (Σ(Si^2^) / K) / [Σ(Xi–X)^2^ / (K-1)] [2–4]. The authors noted that they determined that a sample size of three was sufficient to test the study’s hypothesis. In response to the fifth bullet point, above, the authors noted that in their view non-parametric tests should not be used for sample sizes <10, and that when they reanalysed the available data from the original experiments using one-way ANOVA they obtained statistically significant results supporting dose-dependent and time-dependent effects. A second independent statistical advisor reviewed their reanalyses and indicated that the sample sizes within the groups are too small to allow for an estimation of between-group variation, and that the error bars show within subject variance as opposed to within group variance.

While some image issues were addressed by the original data provided in post-publication discussions, concerns remain about the overall preparation of the figures, statistical analyses and whether the study’s results adequately support the article’s conclusions. As a result, the *PLOS ONE* Editors retract this article.

ZGL, SYN, GMC, PC, and YSL agree with the retraction. JC and ZHG either did not respond directly or could not be reached.
